# Reduced Mimicry to Virtual Reality Avatars in Autism Spectrum Disorder

**DOI:** 10.1007/s10803-016-2930-2

**Published:** 2016-09-30

**Authors:** Paul A. G. Forbes, Xueni Pan, Antonia F. de C. Hamilton

**Affiliations:** 1 Institute of Cognitive Neuroscience, University College London, London, UK; 2 Department of Computing, Goldsmiths, University of London, London, UK

**Keywords:** Mimicry, Virtual reality, Social cognition, Kinematics, Imitation

## Abstract

Mimicry involves unconsciously copying the actions of others. Increasing evidence suggests that autistic people can copy the goal of an observed action but show differences in their mimicry. We investigated mimicry in autism spectrum disorder (ASD) within a two-dimensional virtual reality environment. Participants played an imitation game with a socially engaged avatar and socially disengaged avatar. Despite being told only to copy the goal of the observed action, autistic participants and matched neurotypical participants mimicked the kinematics of the avatars’ movements. However, autistic participants mimicked less. Social engagement did not modulate mimicry in either group. The results demonstrate the feasibility of using virtual reality to induce mimicry and suggest mimicry differences in ASD may also occur when interacting with avatars.

## Introduction

Mimicry involves the unconscious imitation of other people’s behaviour. Research in both social psychology and cognitive neuroscience has demonstrated that mimicry is not only ubiquitous but is also a powerful and versatile tool in everyday social interactions (Chartrand & van Baaren 2009). Individuals who receive a diagnosis of autism spectrum disorder (ASD) have significant impairments in social communication and interaction (American Psychiatric Association 2013) which may include differences in mimicry behaviour (Edwards 2014). The current study’s primary aim was to establish whether any potential differences in mimicry behaviour in ASD could be investigated within a rich and ecologically valid, interactive virtual reality (VR) environment.

### Mimicry as a Social Behaviour

Hamilton (2008) made an important distinction between mimicry and emulation. Mimicry involves implicitly and automatically copying the detailed kinematic features of an observed action, rather than just the action goal. Conversely, emulation involves copying the explicit goal of an observed action. Whilst emulation is useful in practical situations (e.g. when learning how to use a tool), Wang and Hamilton (2012) have argued that mimicry is fundamentally a social behaviour so modulated by social cues in a subtle and sophisticated manner. This has been captured in their social top-down response modulation (STORM) model (Wang and Hamilton 2012). Mimicry has been measured using a range of approaches including naturalistic studies involving live confederates (e.g. Chartrand and Bargh 1999), reaction time tasks using stimulus–response compatibility paradigms (e.g. Brass et al. 2000), and kinematic studies using motion tracking (e.g. Castiello et al. 2002). All these approaches have converged on the finding that a range of social cues, such as attractiveness of the interaction partner (van Leeuwen et al. 2009), eye-contact (Wang et al. 2010), pro-social priming (Leighton et al. 2010; Wang and Hamilton 2013), and, beliefs about the animacy of the interaction partner (Bird et al. 2007; Castiello et al. 2002), modulate mimicry behaviours in neurotypical participants.

### STORMy Interactions: Mimicry in ASD

Autistic people have significant difficulties in everyday social interactions (American Psychiatric Association 2013). Hamilton (2008) suggested that autistic participants perform well on emulation tasks, but tend to perform differently on mimicry tasks compared to neurotypical participants. For example, Hobson & Lee (1999) found that autistic participants were proficient in copying goal-directed actions, but tended not to copy the style with which the experimenter executed those actions. Similarly, McIntosh, Reichmann-Decker, Winkielman & Wilbarger (2006) found that, unlike neurotypical participants, autistic adolescents and adults did not spontaneously mimic happy and angry facial expressions. Yet, when explicitly instructed to copy an observed facial expression autistic participants performed as neurotypical participants. Moreover, Wild, Poliakoff, Jerrison and Gowen (2012) found autistic participants were less sensitive to the duration, velocity and vertical amplitude of observed actions during an imitation task. Eye-tracking also revealed more goal-directed eye-movements in ASD suggesting an over-reliance on goal-directed imitation strategies in ASD and a reduced propensity to mimic. A recent meta-analysis of 53 studies investigating imitation abilities in ASD supported Hamilton’s proposal. It showed spared performance when copying only the goal of an action (i.e. emulation) but impairments when copying both the form (i.e. style) and the goal of an action (Edwards 2014).

The finding that mimicry is different in ASD, a condition characterised by difficulties in social interaction, is in line with Wang and Hamilton’s (2012) proposal that mimicry is fundamentally a social behaviour. It is important to note, however, that Hamilton (2008) has stressed that it is not mimicry *per se* which is impaired in ASD, as autistic children and adults can and do spontaneously copy the actions of others. For example, some autistic individuals display echopraxia characterised by an increased tendency to involuntarily copy the actions of others (Spengler et al. 2010). Rather, it is the top-down social modulation of mimicry that is aberrant in ASD. Hamilton’s hypothesis has been supported by several recent studies using a stimulus–response compatibility paradigm. These show that automatic imitation is intact in ASD as showed by faster responses to congruent rather than incongruent actions, but modulation of this congruency effect by social cues may be atypical. For example, Cook and Bird (2012) showed pro-social priming relative to non-social priming led to an enhancement of automatic imitation in neurotypical participants but not in autistic participants. Similarly, Grecucci et al. (2013) found automatic imitation is enhanced in neurotypical participants, but not in ASD, when preceded by emotional facial expressions. Finally, Forbes, Wang and Hamilton (2016) showed that direct gaze socially modulates mimicry in neurotypical participants but not in ASD.

### Using Virtual Reality to Induce and Modulate Mimicry

A significant limitation of previous studies investigating the social modulation of mimicry in ASD is that they typically displayed isolated hand stimuli within a limited social context and measured participants reaction times to make simple finger movements (e.g. Cook and Bird 2012; Grecucci et al. 2013; Forbes et al. 2016). The current study aimed to create a more ecologically valid mimicry paradigm by creating an interactive two-dimensional (2D) VR environment. Pan and Hamilton (2015) previously found that during a drum tapping game participants displayed a greater tendency to mimic when interacting with a VR avatar compared to a bouncing ball. In their paradigm, a sense of interactivity was achieved by programming the avatar to orient her head to the participant’s head position when it was the participant’s turn to respond. The avatar was also responsive to the participant’s movements as she would wait for the participant to finish their turn before starting her own.

We aimed to combine the VR approach used by Pan and Hamilton (2015) with the kinematic approach used by Wild et al. (2012) to try and induce and socially modulate mimicry in adults with and without a diagnosis of ASD. As VR technologies become more accessible they are increasingly being used to teach and train social skills, such as job interview training, in ASD (e.g. Smith et al. 2014; see; Wang and Reid 2010, for a review). It is therefore important to establish whether the behaviours autistic individuals display in everyday life, such as differences in eye-contact, gesture and joint attention, also occur when interacting with and responding to VR avatars.

To investigate this with regards to mimicry differences, participants played a game with several avatars during which they observed an avatar point to a series of three targets out of a possible four targets on the virtual table in front of them. Participants were given goal-orientated instructions as they were told to point to the same targets the avatar pointed to on the table in front of them. However, the height of the avatar’s movements was manipulated to see whether participants’ own movements were sensitive to the kinematics of the avatars’ movements. Each participant played the game with a socially engaged and with a socially disengaged avatar. The study aimed to explore three questions:

1. Would neurotypical participants mimic the avatar despite being told only to copy the goal of the observed action?
2. If so, would this mimicry be modulated by the social engagement of the avatar?
3. Would there be any differences in mimicry behaviour in ASD?

## Method

### Participants

Twenty-five neurotypical participants and twenty-six autistic participants were recruited from an autism database at the authors’ institution. Groups were matched on age, gender, handedness, and, verbal and performance IQ using either the Wechsler Adult Intelligence Scale (WAIS-III UK; Wechsler 1999a) or Wechsler Abbreviated Scale of Intelligence (WASI-II, Wechsler 1999b; Table 1). Autistic participants had a diagnosis of Asperger’s Syndrome (20), autism (4), or, autism spectrum disorder (2) from an independent clinician.

**Table 1:** A comparison of the autisitc and neurotypical samples

	NT mean (SD)	ASD mean (SD)	ASD vs. NT
N	25	26	–
Left-handed	3	4	–
Female	6	4	–
Age (years)	27.5 (6.1))	28.3 (6.1)	*p* = 0.66
Verbal IQ	123.6 (15.0)	123.2 (13.9)	*p* = 0.91
Performance IQ	115.4 (15.0)	113.2 (12.9)	*p* = 0.58
Fullscale IQ	122.3 (14.5)	120.9 (12.9)	*p* = 0.62
ADOS: comm	–	2.5 (1.3)	–
ADOS: RSI	–	6.0 (1.9)	–
ADOS: total	–	8.6 (2.9)	–

Autistic participants were also tested on module 4 of the Autism Diagnostic Observation Schedule (ADOS-G-Lord et al. 2000) or ADOS-2 (Lord et al. 2012) by a trained researcher with research-reliability status. Seven participants met the ADOS classification for autism, twelve for autism spectrum, and, seven did not meet the classification of autism or autism spectrum. However, all seven who did not meet the cut off for an overall classification of autism or autism spectrum, reached the cut-off for autism spectrum on either the communication or reciprocal social interaction subscale. All participants were financially reimbursed for the time and gave written informed consent to participate. All procedures were approved by the local Research Ethics Committee.

### Materials

The avatars’ pointing movements were animated with pre-recorded motion captured data. These data were recorded using an electromagnetic marker (Polhemus LIBERTY system, Colchester, USA) and mapped onto the avatar using the software packages MotionBuilder (http://www.autodesk.com/motionbuilder) and Vizard (WorldViz Inc, Santa Barbara, USA). During motion capture, a piece of card with markings on it assisted the creation of the high (approximately 11 cm peak height above the table) and low (3 cm) conditions. The speech for the engaged and socially disengaged avatars were recorded from two different female actors.

**Fig. 1:**
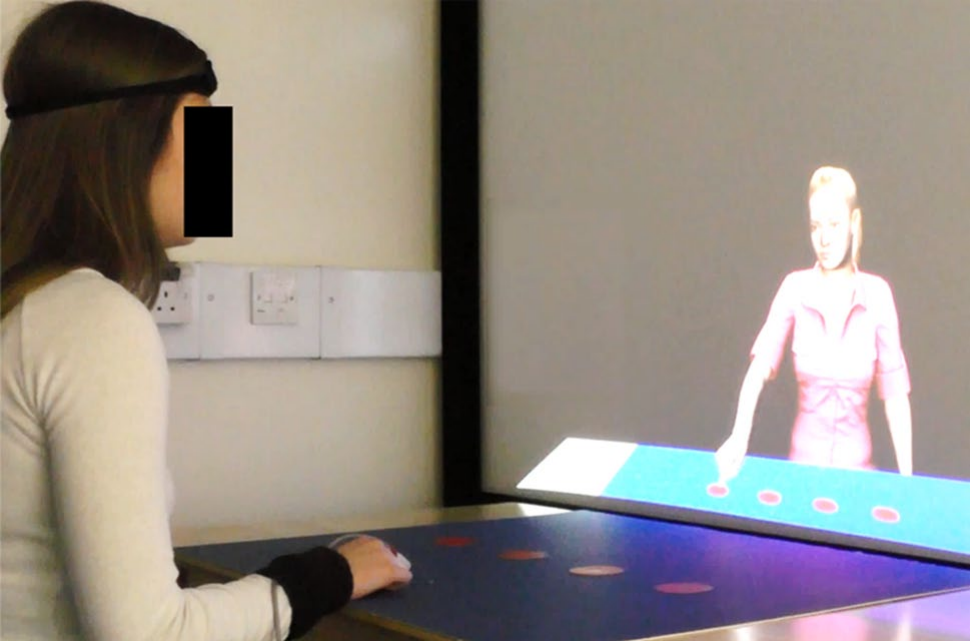
A demonstration of how the virtual world extended into the physical world

Participants sat approximately 70 cm from a 160 × 90 cm projector screen on which the VR graphics were displayed in 2D. An electromagnetic marker (Polhemus LIBERTY system, Colchester, USA) was attached to the top of participants’ right index finger and forehead. The marker on their index finger allowed their finger movements to be recorded, whilst the marker on their forehead allowed the socially engaged avatar to give participant’s eye-contact when smiling at them at the end of each trial. On the table in front of the participants, there was a piece of 81 × 66 cm blue card with four 6 cm diameter red circles stuck in the middle of it. The centre of the circles were 15 cm apart from each other and were 30 cm in front of the participants. These red circles acted at the targets. There was also a 6 × 4 cm piece of blue card stuck 10 cm in front of the participant which acted at the ‘resting pad’ where participants were required to place their right index finger when not moving. The physical world extended into the VR world on the projector screen. Thus, the avatar was also sat at a table with a piece of blue card with four red targets on it (Fig. 1).

### Experimental Design

A 2 × 2 design was used with height (high/low) and engagement condition (engaged/disengaged) as within-subject factors and group (neurotypical/ASD) as a between-subject factor. In each block there were 64 trials (32 high and 32 low) with 16 different movement combinations repeated four times.

### Procedure

Participants came into the lab as part of a research day. Participants were told that they would be playing a game with two avatars, Jessie and Kate, but would first practice the game with another avatar, Mike. Participants were told that the avatars’ movements were based on the movements of people that had previously been in the lab. Before playing the game with Mike, Jessie or Kate, participants completed calibration during which they were required to place their right index finger into the middle of each of the four targets and the resting pad so that their locations could be recorded.

In the practice session with Mike, participants were told that they would hear a ‘dong’ sound which was the avatar’s cue to move. This ‘dong’ sound occurred at the beginning of each trial after a variable delay (1200–1800 ms). The avatar would then point to three of the targets in front of them before returning to their resting position. A ‘ding’ sound then occurred after a variable delay (1200–1800 ms). This sound acted as the participants’ cue to move and they were instructed to point to the same targets that the avatar moved to. Once the participants completed their movements they were instructed to return to their resting pad and this triggered the next trial. The spatial correspondence between the avatars’ and participants’ targets was explained to the participants. For example, if the avatar pointed to the target on her far left, participants should point to the target on their far right. Participants were given approximately 10 practice trails with Mike before the start of the experiment to ensure they understood the task instructions.

**Fig. 2:**
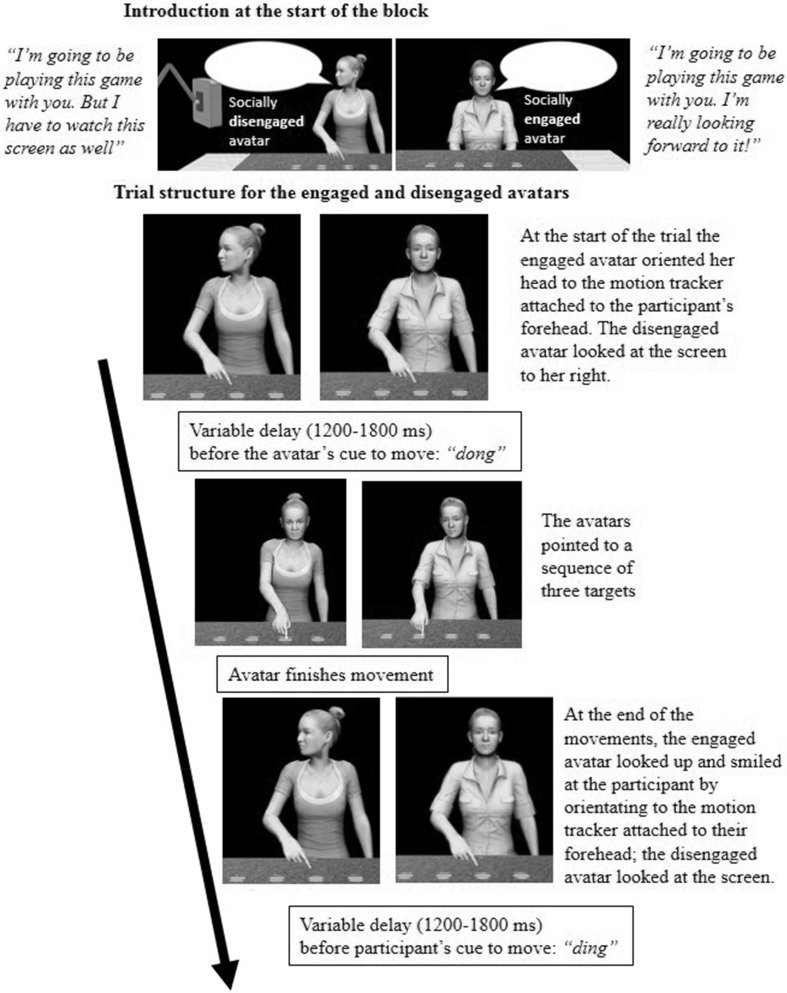
The trial structure for the socially engaged and socially disengaged avatars

Participants then played the game with Kate and Jessie. For each participant, one avatar was socially engaged and the other was socially disengaged (Fig. 2). The order and engagement of the avatars was counter-balanced across participants. Before the game started, the socially engaged avatar said,

“Hi, my names Kate/Jessie and I’m going to be playing this game with you. I’m really looking forward to it” and then smiled at the participant, whereas the social disengaged avatar said,

“Hi, my names Kate/Jessie and I’m going to be playing this game with you. But I have to watch this as well” and then looked away at a virtual monitor on her right hand side. The trial structure was then the same as for the practice session expect that the socially engaged avatar looked up and smiled at the participant and continued to look at them during their response. Conversely, the socially disengaged avatar looked away at the monitor to her right after having completed her movements. So she was not looking at the participant when they made their movements. Finally, in order to measure co-presence, after each game participants were asked to rate on a Likert scale from 1 (not at all) to 7 (very much so): “How much did you behave as if Jessie/Kate were real?”

## Results

### Excluded Data

**Fig. 3:**
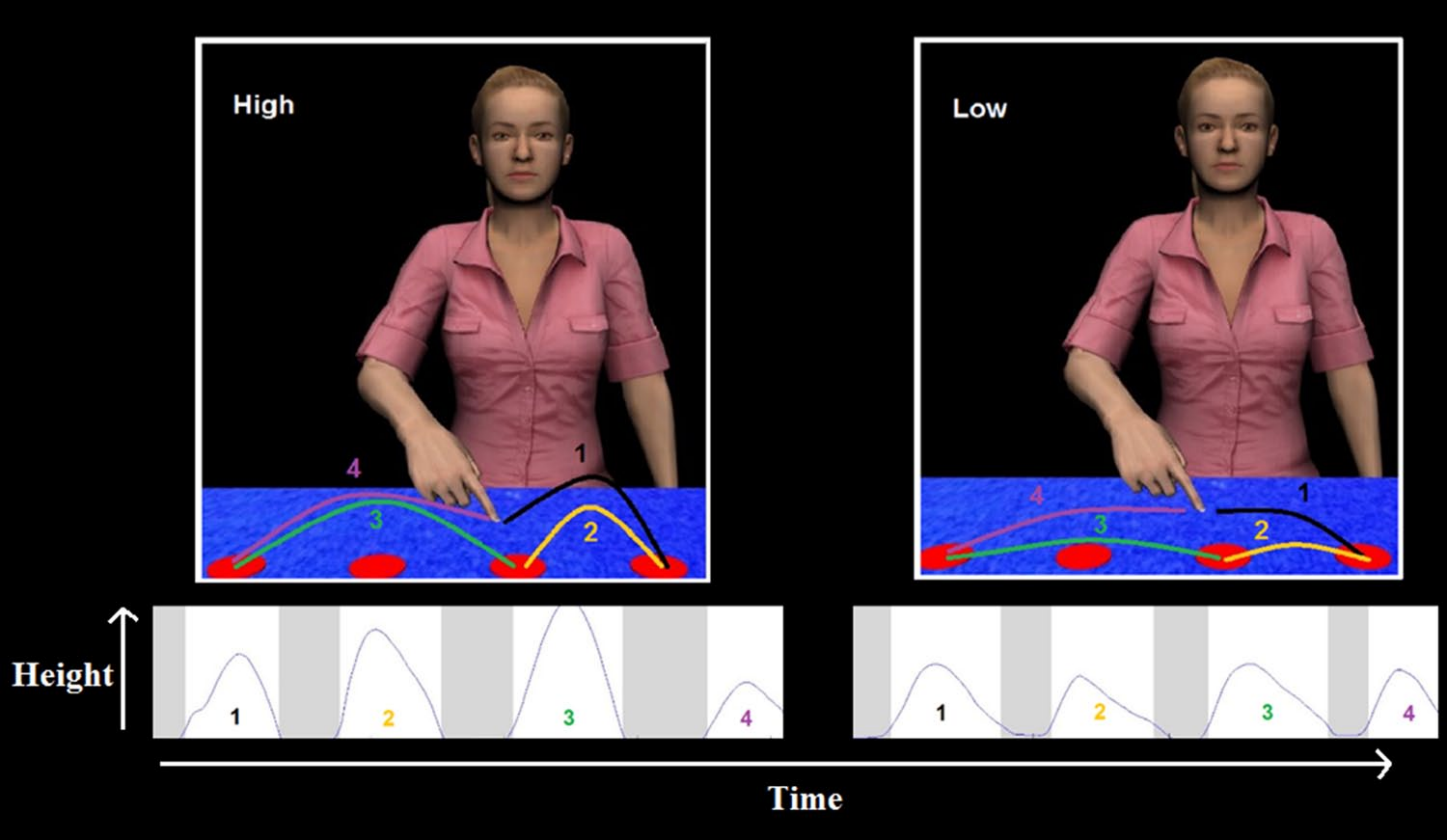
An example of a high and low trial (*above*) and a typical participant movement profile to these observed actions chunked into four movements (*below*). Only movements 2 and 3 were analysed

The movement data were analysed using Matlab R2013b (MathsWorks, Natick, USA). Movement data were filtered with a Butterworth filter to remove high frequencies. Each participant’s calibration data were used to chunk each trial into four movements: (1) the movement to the first target from the resting pad, (2) the movement to the second target; (3) the movement to the third target, (4) the movement back to the resting position (Fig. 3). On 4.27 % of trials, the data could not be chunked into four movements and these were excluded from the analysis. There were no significant differences between neurotypical and ASD in the number of trials that could not be chunked into four movements (Mean (SD): neurotypical 3.66 % (5.35 %); ASD 4.87 % (6.26 %); *t*_49_ = −0.741, *p* = 0.462). On 3.13 % of the trials participants failed to move to the correct targets. There were no significant differences between neurotypical and ASD in the number of incorrect trials per block (Mean (SD): neurotypical 2.31 % (1.93 %); ASD 3.90 % (4.99 %); *t*_32.61_ = −1.516, *p* = 0.139). By combining these two exclusion criteria, the total proportion of trials excluded was 6.62 %. There were no significant differences between the proportion of trials excluded between the two groups (Mean (SD): neurotypical 5.47 % (5.39 %), ASD 7.72 % (7.99 %); *t*_49_ = −1.176, *p* = 0.245).

### Peak Height Analysis

**Fig. 4:**
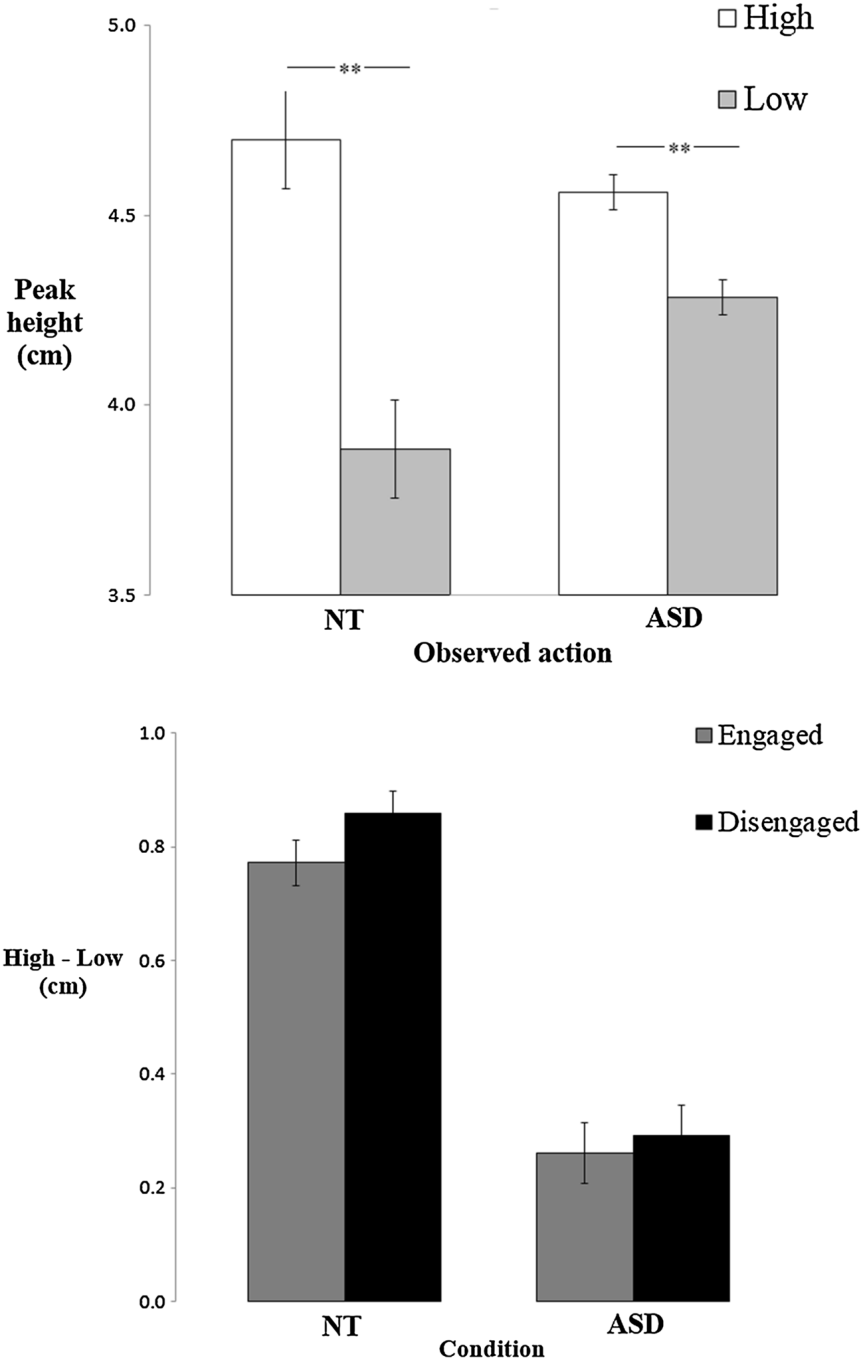
*Top*: mean (+/− SEM) peak heights between the targets in the high and low conditions. *Bottom*: mean (+/− SEM) difference between the high and low conditions for the engaged and disengaged conditions

The mean peak height of the movements between the targets (the mean of movements 2 and 3) for each trial were subject to an ANOVA with engagement condition (engaged/disengaged) and height (high/low) as within-subject factors and group (neurotypical/ASD) as a between-subject factor. This revealed a main effect of height (*F*_1,49_ = 16.28, *p* < 0.001, η_p_^2^ = 0.249). Post-hoc t-test revealed the peak height of participants’ movements were significantly higher having observed the avatar move with a high, compared to low, trajectory between the targets (*t*_50_ = 3.89, *p* < 0.001; Fig. 4 Top panel). This difference between the high and low observed actions was significant for both neurotypical (*t*_25_ = 3.16, *p* = 0.004, *d* = 0.631) and autistic (*t*_25_ = 3.02, *p* = 0.006, *d* = 0.592) participants. There was a marginally significant interaction between height and group (*F*_1,49_ = 3.99, *p* = 0.051; η_p_^2^ = 0.075 Fig. 3 Top panel). Neither the interaction between height and condition, or, height, condition and group were significant (*F* < 0.8; Fig. 4 Bottom panel).

### Co-Presence

**Fig. 5:**
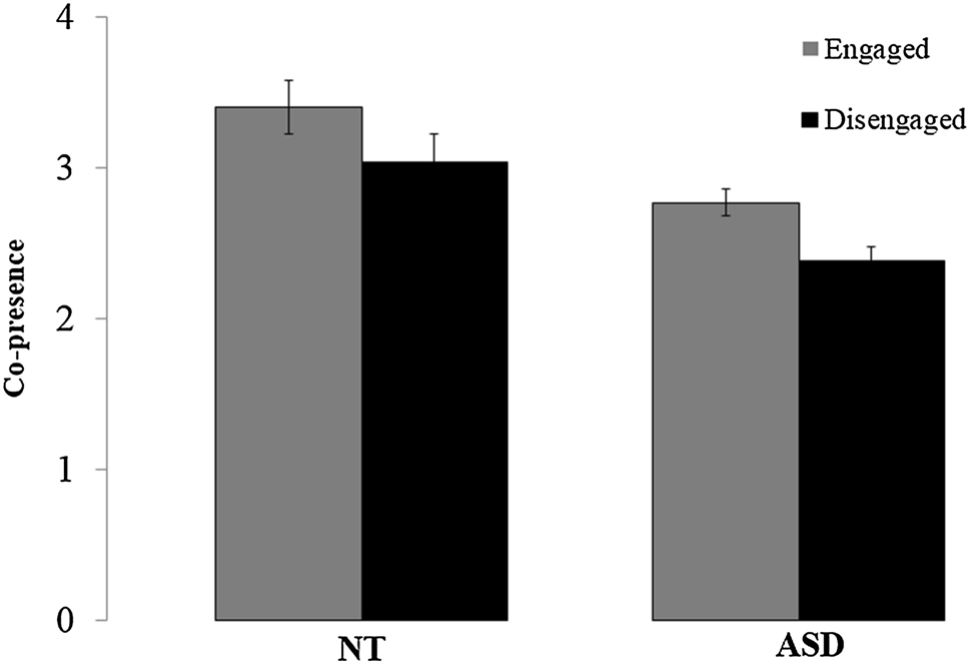
Mean (+/− SEM) co-presence scores

Overall participants’ co-presence ratings were low (Fig. 5). These scores were subject to a 2 × 2 ANOVA with engagement (engaged/disengaged) as a within-subject factor and group as a between-subject factor. This revealed marginal effect of engagement (*F*_1,49_ = 3.54, *p* = 0.066) and group (*F*_1,49_ = 3.21, *p* = 0.079), but no interaction between engagement and group (*F*_1,49_ = 0.004, *p* = 0.951).

## Discussion

The study’s primary aims were to use VR to induce and socially modulate mimicry in neurotypical participants and to explore any differences in ASD. Participants mimicked the kinematics of the avatars’ movements despite being told only to copy the goal of the observed action. Autistic participants tended to mimic but did so to a lesser extent. In neither group, however, was mimicry modulated by the social engagement of the avatar. Possible reasons for this are discussed in further detail below.

### A Novel Paradigm for Inducing Mimicry in VR

The results demonstrate that VR avatars can be used to induce mimicry in both neurotypical and autistic participants. Despite participants being told to point to the same targets the avatar pointed to, they were also sensitive to the kinematics of the observed action, rather than just the action goal. For example, on trials where the avatar moved with a high trajectory between the targets, participants also tended to move with a higher trajectory compared to trials where the avatar moved with a low trajectory. This supports previous kinematics studies, such as that by Wild et al. (2012), in which participants copied the vertical and horizontal amplitude of observed actions despite being given goal-orientated instructions. Previous studies investigating mimicry within a VR setting had only explored reaction time measures of mimicry, such as a stimulus response compatibility paradigm (Pan and Hamilton 2015). The present study extends this work by demonstrating that participants mimic the kinematics of avatars’ movements. More generally, the present study adds to the growing number of studies which highlight the feasibility of VR in the ecologically valid study of human social interaction (Bohil et al. 2011; Georgescu et al. 2014). Our VR paradigm also has the potential to be used in combination with neuroimaging methods, such as functional near infrared spectroscopy, to elucidate the neural underpinnings of mimicry and how these might be different in ASD.

### Reduced Mimicry in ASD

Both the neurotypical and ASD group mimicked the avatars movements, yet autistic participants did so to a lesser extent. This supports previous work demonstrating that autistic individuals can and do under certain conditions spontaneously mimic (Cook and Bird 2012; Grecucci et al. 2013) but there is a reduced propensity to do so (Edwards 2014). Most studies demonstrating a reduced propensity to mimic in ASD investigated children (e.g. Jiménez et al. 2014) and those conducted with adolescences or adults have focused on facial mimicry (Hertzig et al. 1989; McIntosh et al. 2006). Thus, the current study extends this work by showing that this reduced propensity to mimic in ASD continues into adulthood, is not restricted to spontaneous facial mimicry, and, most interestingly, occurs in a VR environment. Importantly, the groups did not differ in terms of their ability to copy the goal of the action (i.e. emulation) as there were no significant differences between the groups in the proportion of trials in which participants pointed to the incorrect targets. Again, this finding is supported by previous work showing intact emulation in ASD (Edwards 2014). Together, these findings support Hamilton’s (2008) proposal of intact emulation yet differences in mimicry in ASD. Finally, the finding that mimicry differences in ASD occur when interacting with VR avatars has important practical and clinical implications for VR training programmes, and, potentially, VR diagnostic tools (Scassellati 2007). It suggests that the behaviours autistic individuals display in everyday life also occur when interacting with and responding to VR avatars. Although limitations of our current VR approach are discussed below.

### Unmodulated Mimicry: Co-Presence and Social Cues

Mimicry was not modulated by how socially engaged the avatar was in either neurotypical or autistic participants. This is at odds with STORM and a series of previous studies which demonstrated that social cues, such eye-contact (Forbes et al. 2016), pro-social priming (Cook and Bird 2012) and emotional facial expressions (Grecucci et al. 2013), modulate mimicry in neurotypical participants; yet, this modulation is reduced in ASD. There are several possible reasons as to why the social manipulation did not modulate mimicry in the current study. Wang and Hamilton (2012) proposed that the effect of eye-contact on mimicry is mediated by an audience effect, whereby the enhancement occurs when participants feel the observer is maintaining social engagement with them throughout the response period. In the current study, the socially engaged avatar gave participants eye-contact throughout their response period so it is unclear why mimicry was not enhanced. One possible reason could be the lack of co-presence with the VR avatars; mean co-presence scores were low. Thus, if participants felt the avatars were unrealistic this may have nullified the impact of any social manipulation and caused low co-presence scores. The avatars’ hand movements were motion captured so based on those of a human. This may account for the reliable mimicry effect as participants are likely to have regarded these movements as realistic. However, the avatars’ head movements, and facial expressions, such as the socially engaged avatar’s smile, were key frame animated. Although, participants’ qualitative experiences towards the avatars were not collected in the current study, in previous VR studies participants have reported that the avatars “were slightly robotic without facial expression which lessened impact” (Pan et al. 2016, p. 11). Moreover, Moser et al. (2007) have highlighted differences in neural activation, such as reduced activation of the fusiform gyrus, when viewing an avatar with emotional facial expressions compared to a human face displaying the same expressions. Thus, the present limitations of the VR, especially with regard to realistic facial expression, may have accounted for the lack of co-presence and the lack of social modulation in the present study.

The 2D nature of our VR environment may also have contributed to the low co-presence scores. Although the physical world of the participant continued into the virtual world on the screen in front of them, there was a tangible divide between the physical world of the participant and the virtual world of the avatar. Schultze (2010, p. 439) has highlighted how “one key-determinant of co-presence is … to jointly manipulate shared space and shared objects.” Therefore, the current paradigm may benefit from being implemented in a fully immersive VR setting, for example using a head-mounted display (HDM), such as the Oculus Rift or HTC Vive. This would allow the participants to be embodied (i.e. have their own avatar) and share the virtual space with the avatar, for example, both avatar and participant could point to the same virtual targets. However, studies using such an approach typically have the virtual targets positioned in mid-air without a table, but the kinematics of movements to such targets might differ. Implementing our paradigm safely and effectively using a HMD *with* a physical table is technically challenging. A failure to embody participants accurately within a fully immersive HMD runs the risk of participants injuring their fingers on the table in front of them when pointing to the targets.

There was some level of interaction between the avatar and participant in the current study. For example, the avatar did not start her turn until the participant had returned to the resting pad, and, after the engaged avatar had finished her turn she oriented to a motion tracker attached to each participant’s forehead thereby giving a sense of eye contact. Despite these advantages over simple video stimuli, participants were still watching animations on a screen in front of them. Reader and Holmes (2015) directly compared real life and video stimuli during an imitation task and found reduced object-directed imitation accuracy with the use of video stimuli. Furthermore, reduced activation of human motor cortex has been found when observing motor acts in videos compared to live movements (Järveläinen et al. 2001). Again, the use of a fully immersive, 3D environment, or, the use of real-life interaction partners may result in the social modulation of mimicry within the current paradigm.

### Unmodulated Mimicry: Timing and Task Demands

In studies investigating social modulators of mimicry within a stimulus—response compatibility paradigm, there is usually a small time window between the social manipulation, the observed action and the subsequent response. For example, in Forbes et al. (2016) the delay between the social manipulation and observed action was either 200 or 800 ms. Participants were then required to respond as soon as they saw the actor’s hand move in the video. Similarly, in Grecucci et al. (2013) the facial expression was presented for 500 ms, participants then observed the moving hand for 1105 ms before being required to respond. Finally, in Pan and Hamilton (2015; Experiment 2) the interaction between form (avatar vs. ball) and congruency (i.e. mimicry) was only found on reaction times to tap the first, but not the last, drum in the sequence. Together these studies support the view that for certain social manipulations the delay between action observation and performance needs to be minimised in order for the social manipulation to modulate mimicry. Future studies investigating social modulators of mimicry within the present paradigm may benefit from comparing the kinematics of movements to the first target.

The relatively high tasks demands in the current study may have contributed to a lack of social modulation. Error rates in stimulus–response compatibility paradigms are typically less than 0.1 % (e.g. Wang et al. 2010; Bird et al. 2007). In Pan and Hamilton’s (2015) task mean error rates were between 1.2 and 1.5 %. In the present study the error rate was approximately double this for the neurotypical participants (2.6 %). The lower error rate in Pan and Hamilton (2015) is likely due to the lower memory demands of their task. The required drum sequence was displayed on a virtual tablet in front of the avatar, whereas, in the current study participants had to memorise the correct three target sequence. Thus, the higher task demands in the present study may have nullified any potential social modulation of mimicry. Finally, it is also possible that lower task demands will enhance mimicry as this could increase participants’ ability to process the motion of the avatar’s movements (Rees et al. 1997). Future studies could reduce the task demands by having participants point to fewer targets.

## Conclusions

To conclude, we provide a novel paradigm which enables mimicry to be induced in a rich and ecologically valid, interactive VR environment. Participants copied the kinematics of the avatars’ movements, despite being instructed only to copy the goal of the observed action. The study reinforces Hamilton’s (2008) proposal of intact emulation but differences in mimicry in ASD as autistic participants showed reduced mimicry compared to neurotypical participants. The findings have implications for VR training programmes and also potential VR diagnostic tools, as it suggests that behaviours autistic people display in everyday life also occur when interacting with avatars. Unlike previous studies investigating the modulation of mimicry, the social manipulation in the present study failed to modulate mimicry. There are several possible reasons as to why the social manipulation did not modulate mimicry, including the timing of the manipulation and the present limitations of facial expressions in VR. Future studies should explore these possibilities.
